# Tumor-associated macrophage expression of interferon regulatory Factor-8 (IRF8) is a predictor of progression and patient survival in renal cell carcinoma

**DOI:** 10.1186/s40425-019-0630-0

**Published:** 2019-06-20

**Authors:** Jason B. Muhitch, Nicholas C. Hoffend, Gissou Azabdaftari, Austin Miller, Wiam Bshara, Carl D. Morrison, Thomas Schwaab, Scott I. Abrams

**Affiliations:** 1 Department of Urology, Roswell Park Comprehensive Cancer Center, Buffalo, New York USA; 2 Department of Immunology, Roswell Park Comprehensive Cancer Center, Buffalo, New York USA; 3 Department of Pathology, Roswell Park Comprehensive Cancer Center, Buffalo, New York USA; 4 Department of Biostatistics and Bioinformatics, Roswell Park Comprehensive Cancer Center, Buffalo, New York USA

**Keywords:** Interferon regulatory factor-8, Renal cell carcinoma, Macrophages, Tumor progression

## Abstract

Tumor-associated macrophages have been well-characterized in solid malignancies, including renal cell carcinoma and generally correlate with poor prognosis. However, the molecular mechanisms which govern intratumoral macrophage behavior and patient outcome are unclear. Here, we investigated whether alterations in macrophage expression of the transcriptional regulator for myeloid commitment and function, interferon regulatory factor-8 (IRF8), could predict survival of clear cell renal cell carcinoma patients. Transcriptional analysis of publicly available data revealed elevated IRF8 expression was associated with prolonged disease-free survival. Evaluation of protein expression within histologic sections of primary clear cell renal cell carcinoma patient samples showed intensity of IRF8 by CD68^+^ macrophages correlated inversely with stage. Survival outcomes of patients with primary or metastatic disease could be stratified on the basis of IRF8 levels by macrophages. Patients with high levels of IRF8 expression within metastatic sites had prolonged overall survival (log-rank *P* < 0.01, HR = 0.44, 95% C.I.: 0.23–0.84) compared to patients with low levels of IRF8 expression. When patient cohorts were further separated based on macrophage infiltration within metastatic lesions, patients with a macrophage^lo^ IRF8^hi^ profile had a more than 10 year increase in median overall survival compared to patients with a macrophage^lo^ IRF8^lo^ profile (log-rank, *P <* 0.001). In summary, we report that macrophage expression of IRF8 is inversely correlated with tumor mass and directly related to survival outcome. These findings support the utilization of IRF8 expression by macrophages to predict patient outcome, which may have important implications for guiding treatment decisions for renal cell carcinoma patients with metastatic disease.

**Electronic supplementary material:**

The online version of this article (10.1186/s40425-019-0630-0) contains supplementary material, which is available to authorized users.

## Introduction

The clinical behavior of metastatic renal cell carcinoma (RCC) can vary significantly from patient-to-patient, which complicates the tracking of disease progression and designing treatment paradigms. Biomarkers that correlate with progression are likely to improve management by providing indicators of tumor behavior, as well as potential targets for therapeutic intervention. To date, most identified biomarkers reflect tumor-intrinsic properties within the heterogeneous tumor microenvironment (TME) that, depending on which region is evaluated, may or may not express the biomarker [1]. Stromal components that interact directly with the TME may present a more reliable representation of the aggressiveness of malignant disease. Indeed, recent findings have demonstrated that the stromal elements of the TME play a significant role in progression, response to therapy, and even prognosis at time of diagnosis [2].

In diverse solid tumor types, including RCC, tumor-associated macrophages (TAMs) reside within the TME in large numbers and generally correlate with poorer outcomes [3]. Conversely, infiltration of macrophages characterized by an antitumor phenotype has been correlated with improved survival [4]. The complexity of TAM-based prognosis is supported by recent mass cytometry findings that have characterized 17 subtypes of TAMs within clear cell (cc) RCC [5]. In that study, the increased infiltration of 2 macrophage subsets in concert with a reduced accumulation of a third subtype predicted progression-free survival [5]. Thus, the relationship between infiltration and prognosis may reflect the balance between immunosuppressive versus immune-activating subpopulations of intratumoral macrophages. Compared to bulk infiltration, evaluation of TAM behavior may therefore provide a more reliable method to predict outcome. The capacity of TAMs to transition from one functional state to another depends upon the cytokine milieu [6], which may influence their functional contributions. These changes are generally thought to be governed by distinct transcription factors that act as master regulators of cellular identity and function.

Interferon regulatory factor-8 (IRF8) is a myeloid-dependent transcription factor that is indispensable for myeloid commitment and adaptive immunity through its ability to control: *1)* the development of monocytes/macrophages and dendritic cells into productive antigen-presenting cells (APCs) [7]; *2)* the production of pro-inflammatory cytokines, such as IL-12, from APCs [8] which promotes the differentiation of CD4^+^ T_h_1 and CD8^+^ cytotoxic T cells and activation of NK cells, that in turn secrete IFN-γ, an important component of antitumor immunity [2]. Expression of IRF8 in the human myeloid compartment is essential for the development of adaptive immunity, whereby patients with mutations in IRF8 harbor significant deficiencies in circulating monocytes or dendritic cells [7]. Moreover, recent work from our laboratory has identified IRF8 as a previously unrecognized negative regulator of myeloid-derived suppressor cells (MDSCs), which are known to potently inhibit innate and adaptive immunity [9]. In addition to its effects on lineage commitment, in vitro functional studies have demonstrated that IRF8-deficiency within human and murine macrophages abrogates IL-12p40 production in response to IFN-γ [7, 10].

While IRF8 acts as a regulator of lineage commitment and macrophage responses to pathogens [10, 11], less is known regarding the importance of IRF8 as a transcriptional marker of TAM behavior and disease progression in human malignancies. Recent studies performed in our laboratory demonstrate that IRF8 expression in macrophages is important for an anti-metastatic program in preclinical models of mammary cancer and melanoma [12]. We extended these studies here to address whether the level of IRF8 in nephrectomy and metastatic tissues from ccRCC patients could be correlated with disease progression. These studies evaluated IRF8 expression by TAMs and provide the first evidence that protein expression of this transcription factor is decreased in advanced stage patient specimens and can be used to predict long-term survival in a subset of ccRCC patients.

## Material and methods

### TCGA data analysis

The results reported here, in part, are based upon data generated by The Cancer Genome Atlas (TCGA) Research Network: https://www.cancer.gov/tcga. ccRCC patient data including clinical and gene expression profiles were obtained from TCGA and cBioPortal. Only patients with complete clinical data were used in analysis. TAMs (defined by M0 macrophages, *P* value ≤0.1) were determined following deconvolution of TCGA data by CIBERSORT analysis [13]. IRF8 and TAM hi vs low cutoffs were determined using the tertile method, wherein values > 66% expression were considered high and values < 33% expression were considered low; values contained within 33–66% were censored. Correlations with outcome were performed using the survival package in R studio (https://cran.r-project.org/web/packages/survival/index.html).

### Tissue microarray and immunohistochemistry

This retrospective study was performed using tissues that required informed consent for donation. Patient materials selected for tissue microarray (TMA) construction were from those with histologically confirmed RCC and no known prior oncologic treatments. H&E sections were reviewed by pathologists who selected representative areas of RCC. Attention was made to exclude areas of extensive necrosis and hemorrhage within tumors. TMAs were constructed from 0.6 mm tissue cores from formalin-fixed, paraffin-embedded blocks and arrayed into a new recipient paraffin block. A previous study showed that 3 to 4 cores from each sample gave optimal statistical results [14]; therefore TMAs were constructed using 3 cores from each sample.

4-μm sections were cut, placed on charged slides, and dried at 60 °C for 1 hour. Slides were cooled to room temperature, deparaffinized in xylene, and rehydrated using graded alcohols. Antigen retrieval was performed using citrate buffer (BioCare Medical, catalog # CB910) for 60 min in a steamer and cooled for 20 min. Peroxidase was quenched with aqueous 0.3% H_2_O_2_ for 10 min. Slides were loaded on an autostainer and serum-free protein block (DAKO, X0909) was applied for 5 min. The primary antibody ICSBP (Santa Cruz, sc-6058) was applied at 1/400 (goat IgG) for 1 hour, followed by Bio-2-Gt (Vector Labs, PK-6105) for 30 min. Elite ABC (Vector Laboratories, PK-6100) was applied for 30 min followed by DAB chromogen (Dako, K3468) for 5 min. Serum-free protein block was applied again for 5 min. The second primary antibody, CD68 (Dako, M0814), was applied for 1 h at 1/1321 (mouse IgG1) followed by DakoLink for 30 min and DakoEnhancer for 30 min. Fast Red (Agilent, K5355) was applied for 10 min. CD3 staining was performed at 1/100 (Dako, A0452), followed by biotinylated anti-rabbit (Vector Laboratories, BA-1000), then Elite ABC, and finally DAB. Slides were counterstained with Hematoxylin, dehydrated, cleared, and cover-slipped.

#### Pathologic analysis

Studies were performed in accordance with de-identified IRB-approved protocols. The sections were evaluated by two pathologists for specificity of staining. The percentage of cells expressing the marker (reported from 0 to 100 in deciles), and the intensity of expression (reported on a scale from 0 to 3) within CD68^+^ macrophages were quantified in a blinded manner by a board certified GU pathologist (G.A.). The intensity of IRF8 and CD68 staining were scored from 1 to 3 and the quantity of their expression was scored in percent positive cells of all stromal cells in the section. While the TMA consisted of additional renal tumor histologies, only ccRCC specimens were analyzed.

The Biomedical Data Science Department provided all demographic and clinical data associated with the TMAs in a de-identified manner.

#### Selection of IRF8 and TAM cutoffs and statistical analysis

Cutoff values for IRF8 intensity and CD68^+^ TAM infiltration were determined using median values from primary samples. Statistical analyses were performed using GraphPad version 7.0. Results were compared using an unpaired Kruskal-Wallis or Mann-Whitney test, as indicated. Patients without staging information were removed from analysis. Progression-free and overall survival information was available for all patients who had specimens within the TMAs and estimated via the Kaplan-Meier method. Comparisons between groups were performed using the log-rank test. *P*-values less than 0.05 were deemed significant.

## Results

### Transcriptional analysis of IRF8 expression and macrophage infiltration

**Fig. 1:**
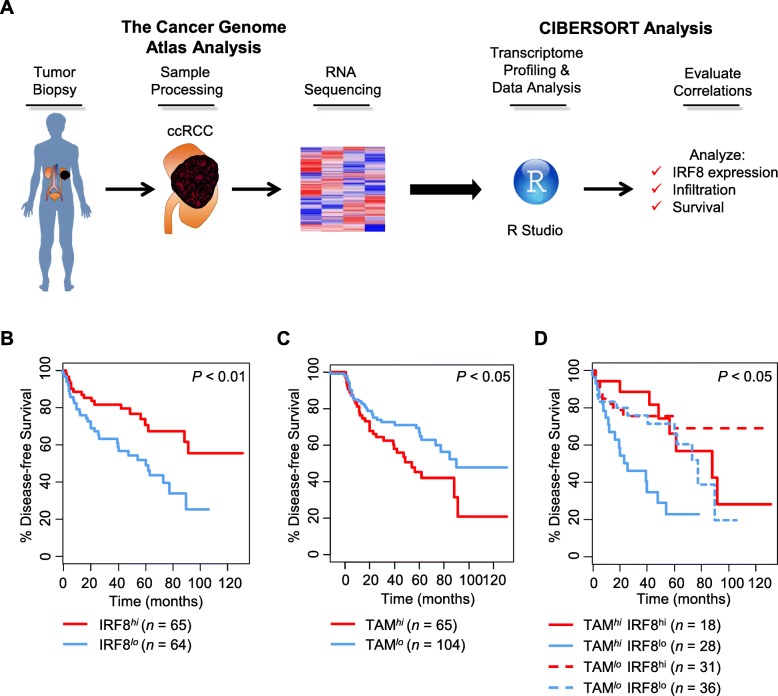
Increased IRF8 transcriptional levels are associated with improved disease-free survival in renal cell carcinoma. **a** Transcriptional analysis of IRF8 expression and macrophage infiltration was performed from TCGA data. Kaplan-Meier plot of survival by IRF8 transcriptional status (**b**). Patients with high levels of IRF8 expression had prolonged disease-free survival (*P* < 0.05 log-rank test). Disease-free survival of patients based on macrophage infiltration (as determined by deconvolution of TCGA data through CIBERSORT) (**c**) and combined macrophage and IRF8 expression (**d**). Patients with high levels of TAM infiltration and IRF8 had significantly better disease-free survival than patients with high levels of TAM and low expression of IRF8 (*P < 0.05 log-rank test). IRF8 and TAM scores were stratified based on a tertile method

To determine whether IRF8 expression in association with TAM infiltration carried prognostic value for patient outcome, transcriptional analysis of TCGA data was initially performed. We focused our analysis on ccRCC to reduce variability of immune infiltration associated with diverse disease types [15]. TCGA data were analyzed using R studio and CIBERSORT to identify intratumoral expression of IRF8 transcripts and to estimate macrophage infiltration, respectively, in patients (Fig. 1a). Based on availability of outcome data, we evaluated disease-free and overall survival and found that patients with high levels of IRF8 expression had longer disease-free survival than those with low IRF8 expression (Fig. 1b). No significant difference was observed for overall survival between patients with high and low levels of IRF8 (Additional file 1: Figure S1A). We used CIBERSORT deconvolution of TCGA data to identify patients with low and high levels of TAMs. Although no difference in overall survival was observed based on TAM infiltration (Additional file 1: Figure S1B), we found that patients with low levels of TAMs had prolonged disease-free survival (Fig. 1c). Combining these transcriptional comparisons of TAM infiltration with IRF8 expression, patients with high levels of both macrophage infiltration and IRF8 expression had significantly longer survival compared to patients with high levels of TAMs and low IRF8 expression (Fig. 1d).

### IRF8 expression within primary RCC specimens

**Fig. 2:**
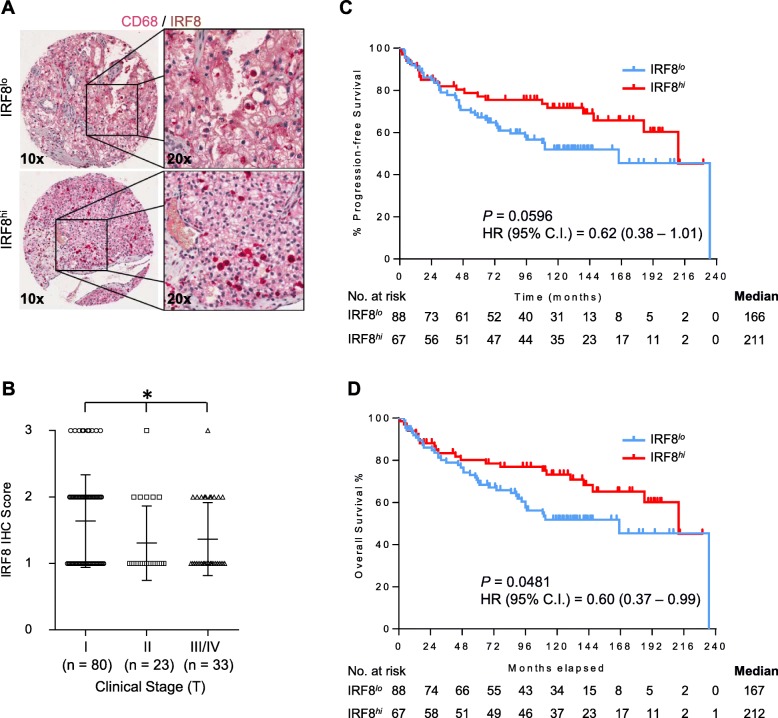
Higher intratumoral IRF8 protein levels are associated with early stage renal cell carcinoma and prolonged survival. **a** Representative primary RCC cases showing different levels of IRF8 protein expression (upper image IRF8^lo^, lower image IRF8^hi^). (**b**) IRF8 protein expression plotted by clinical stage (T). *P < 0.05 (Kruskal-Wallis test). Data for progression-free survival and overall survival are shown according to stratification based on IRF8 status within primary tumors of RCC patients (panels **c** and **d**, respectively). RCC patients with high levels of intratumoral IRF8 (≥ 2 score) had significantly prolonged overall survival than patients with low levels of IRF8 (< 2) based on log-rank test

The observed prolonged survival of patients with elevated levels of IRF8 transcripts and estimated macrophage content (Fig. 1) supports our hypothesis that TAM expression of IRF8 can predict patient survival. However, IRF8 expression can emanate from additional tumor-resident populations, including human tumor cells [16] and therefore our initial findings could be influenced by TAM-independent factors. To specifically address the influence of IRF8 expression within TAMs on outcome, we co-stained primary human ccRCC nephrectomy samples with IRF8 and CD68 to detect expression of IRF8 by TAMs. The clinical characteristics of the 155 ccRCC patients (94 males and 61 females) who had primary nephrectomy samples contained within our cohort are summarized in Table 1. Median age was 58.6 years with patients having a median survival of 212 months. Based upon availability of data, we analyzed progression-free and overall survival as parameters of outcome. IRF8 staining was highly specific and localized to the nucleus of CD68^+^ TAMs (Fig. 2a). In this data set, we found no association between CD68^+^ accumulation and clinical stage, progression-free survival or overall survival (Additional file 1: Figure S2A-C).

**Table 1:** Characteristics of clear cell RCC patients utilized in analysis of primary tumor expression of IRF8

Characteristic	Overall
Participants, *n*(%)	155 (100)
Age at diagnosis, yr (mean ± SD)	58.6 ± 12.8
Gender, *n*(%)	
Male	94 (60.6)
Female	61 (39.4)
Pathological stage, *n*	
I	80
II	23
III	31
IV	2
Unknown	19

We next addressed whether the intensity of IRF8 expression by TAMs was associated with stage or outcome. We observed increased IRF8 intensity (score) by CD68^+^ TAMs in stage I patient tumors (Fig. 2b). We found no association between IRF8 intensity and tumor grade (Additional file 1: Figure S2D). Comparisons of IRF8 scores with survival data showed a trend towards improved progression-free survival with high levels of IRF8 expression that did not reach significance (*P* = 0.0596, Fig. 2c). Elevated IRF8 protein in primary disease was associated with an increase in overall survival (*P* = 0.0481, Fig. 2d). We found no difference in overall survival when comparing outcomes of stage I patients with low versus high IRF8 expression (*P* = 0.2637, Additional file 1: Figure S3).

### IRF8 expression within tumor metastases and correlations with patient survival

**Fig. 3:**
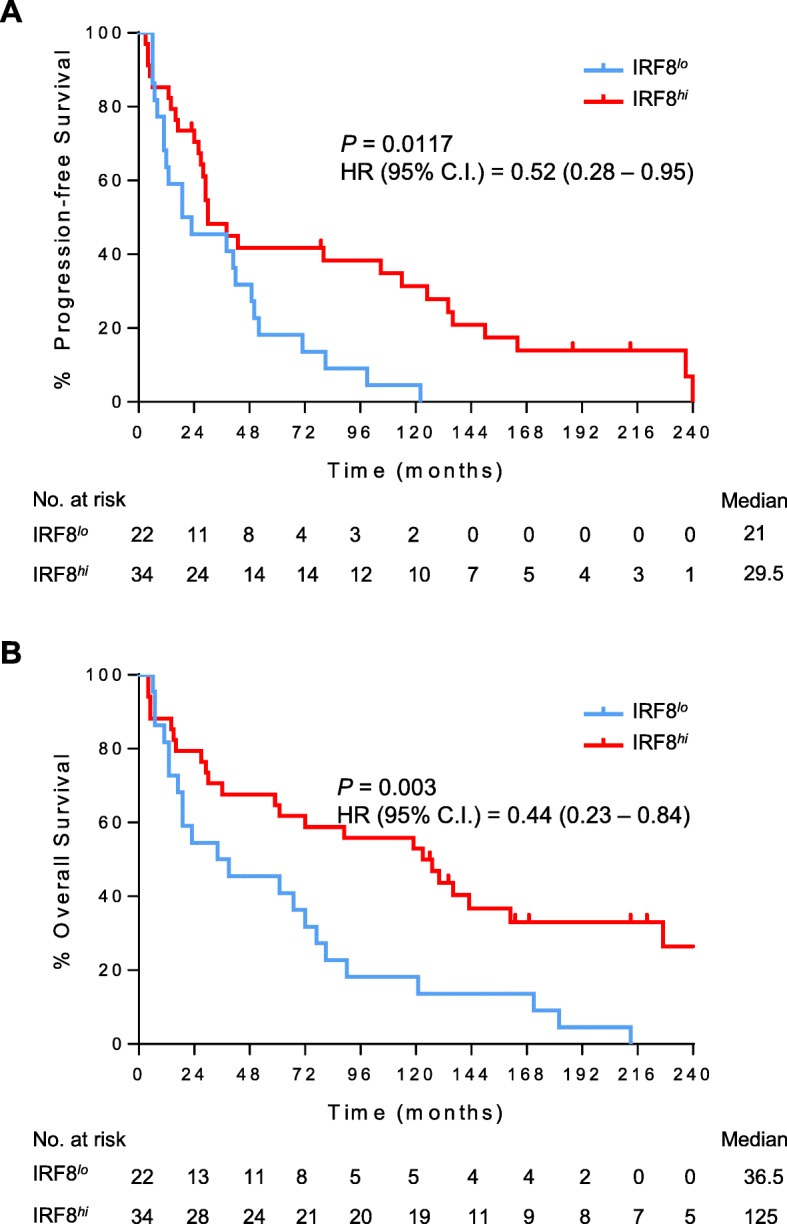
Expression of IRF8 within metastatic renal cell carcinoma tumors is associated with improved patient survival. Kaplan-Meier plots of (**a**) progression-free survival and (**b**) overall survival by IRF8 status within renal cell carcinoma patient metastasis. Patients with high levels of IRF8 (score ≥ 2) expression within metastasis had prolonged progression-free and overall survival compared to patients with IRF8 low (score < 2) metastatic tumors (log-rank test). IRF8 groups were stratified using median cut-off of IRF8 score

Histologic analysis of primary RCC has been utilized for the development of prognostic biomarkers for patient survival, as well as outcomes following treatment with immunotherapy [17, 18]. However, metastatic RCC represents advanced disease and median survival can be less than 2 years [19]. The status of IRF8 and TAMs within metastatic lesions therefore may be a more relevant prognostic indicator of outcome. A few studies have evaluated metastatic tissue for prognostic markers [17], but to the best of our knowledge there has been no investigation into the status of TAM infiltration, as well as transcriptional regulators of macrophage biology including IRF8 within metastatic RCC. A separate TMA was composed consisting of tissue from 56 metastatic tumors from 35 male and 21 female patients diagnosed at an average age of 55.6 years with additional features regarding metastatic location shown in Table 2. Analysis of IRF8 expression by CD68^+^ TAMs within metastatic ccRCC samples showed prolonged progression-free survival of patients with high expression of IRF8 (Fig. 3a). Patients with high levels of IRF8 by TAMs within metastatic lesions had an overall survival advantage of more than 80 months (*P* < 0.01, Fig. 3b).

**Table 2:** Characteristics of clear cell RCC patients utilized in analysis of metastatic expression of IRF8

Characteristic	Overall
Participants, *n*(%)	56 (100)
Age at diagnosis, yr (mean ± SD)	55.6 ± 9.0
Gender, *n*(%)	
Male	35 (62.5)
Female	21 (37.5)
Location, *n*	
Lung	17
Lymph Node	10
Brain	4
Other	25

**Fig. 4:**
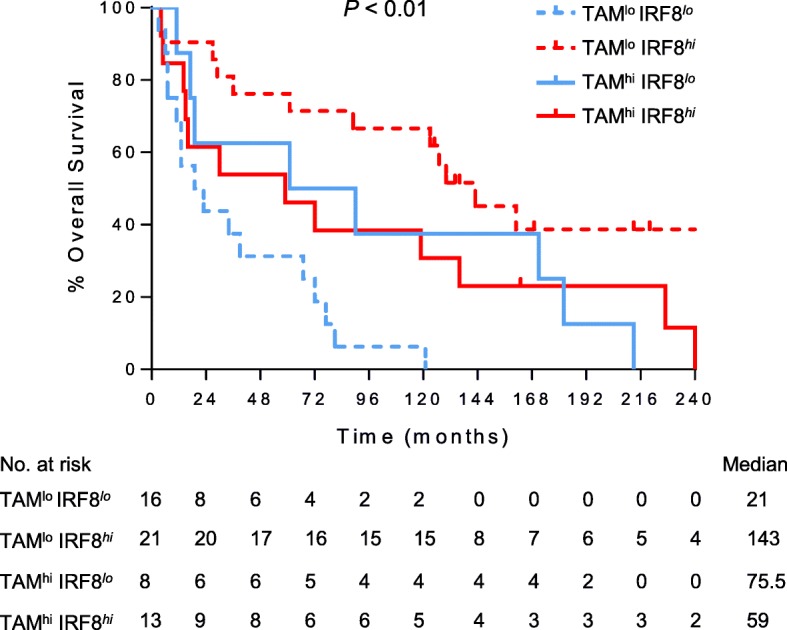
Overall survival is associated with IRF8 expression in metastatic tumors with high or low levels of macrophage infiltration. Kaplan-Meier plot of overall survival by macrophage infiltration, identified by CD68 expression, and IRF8 status within metastasis of renal cell carcinoma patients. Groups were stratified using median cut-off scores for macrophage infiltration (30% of evaluated tumor area). Metastatic patients with low levels of TAM and IRF8^hi^ expression had a more than 10 year increase in median overall survival compared to patients with low TAM and IRF8^lo^ phenotype (log-rank *P* < 0.001, HR = 0.2485, 95% C.I.: 0.1052–0.5869)

Our analysis revealed no difference between TAM infiltration in metastatic tumors expressing high versus low levels of IRF8, and overall survival was not influenced by TAM infiltration (Additional file 1: Figure S4A-B). However, when patients were further stratified according to the frequency of CD68^+^ TAMs and IRF8 expression, patients with low levels of TAMs and high IRF8 expression had a significantly improved survival outcome compared to those who had low levels of TAMs and low IRF8 expression (median overall survival of 143 month to 21 months, respectively, Fig. 4). Metastatic tissues containing low levels of TAMs and IRF8 expression were also correlated with lower levels of CD3^+^ T cells (Additional file 1: Figure S5A-B).

## Discussion

RCC is one of the most heavily immune infiltrated human tumor types [20] with lymphocytes and macrophages representing approximately 80% of intratumoral immune cells [5]. Interestingly, while ccRCC also has the highest level of T cell infiltration and cytolytic activity compared with more than 18 human tumor types [5, 21] and is responsive to multiple classes of immunotherapy [18, 21], T cell infiltration is not a prognostic indicator of survival [22]. Rather, foundational studies have shown a high proliferative capacity of intratumoral T cells is associated with survival [23], signifying that behavior rather than quantity of the infiltrate is a more reliable prognostic indicator.

Here, we evaluated the expression of a key transcription factor of monocyte/macrophage development and function, IRF8 [7, 9, 11] in TAM-based prognosis of patients with ccRCC. The characterization of TAMs in RCC has been performed multiple times with groups using as many as 30 markers to distinguish 17 different subpopulations of TAMs within human RCC [5]. However, there has been little investigation of TAM expression of master transcriptional regulators, including IRF8, in human disease and whether such regulators correlate with disease status and survival.

We found that the intensity of IRF8 staining on TAMs within primary ccRCC was correlated with pathologic T-stage. Evidence that TAMs from larger primary RCC patient tumors produce more immunosuppressive cytokines on a per cell basis [24] than TAMs from smaller tumors further supports the hypothesis that the function, not quantity, of TAMs can influence disease aggressiveness. Our key findings that IRF8 expression by TAMs within both primary and metastatic disease was a better prognostic indicator of survival than TAM infiltration suggests that the behavior of TAMs can be represented by the status of master or major transcriptional regulators. Functionally, TAMs can also directly or indirectly influence infiltration and differentiation states of other key leukocyte subsets, including effector T cells that can promote local anti-tumor responses [5].

A potential limitation of this study, however, is that the analysis was based on conventional pathologic microscopic evaluation, compared to digital methods. However, our reasoning reflected the observation that macrophages within ccRCC displayed diverse morphologic shapes, potentially making it problematic for accurate digital assessment.

In summary, our study supports the notion that TAM-based prognosis carries greater clinical merit if the expression of transcription factors, which provide important insights into functionality, is assessed as part of a broader cellular signature.

## Additional file

Additional file 1: **Figure S1.** Survival of RCC patients by IRF8 and TAM infiltration. **Figure S2.** Stage, progression-free and overall survival are not associated with macrophage infiltration in nephrectomy specimens. **Figure S3.** Overall survival of stage I RCC patients by IRF8 expression in nephrectomy specimens. **Figure S4.** CD68^+^ TAM infiltration is unchanged in metastasis expressing high and low levels of IRF8. **Figure S5.** CD3 T cell infiltration in metastatic RCC tumors. (PDF 563 kb)

## Data Availability

Reagents used in this study were commercially available. TCGA data used to generate survival data is publicly available and no other large data sets were utilized in this study.
