# Supplementary material for: Tumor-associated macrophage expression of interferon regulatory Factor-8 (IRF8) is a predictor of progression and patient survival in renal cell carcinoma
Source: J Immunother Cancer. 2019 Jun 20;7:155. doi: 10.1186/s40425-019-0630-0 (PMC6585080; doi:10.1186/s40425-019-0630-0)
Supplement: Supplementary file 1 — Figure S1. Survival of RCC patients by IRF8 and TAM infiltration. Figure S2. Stage, progression-free and overall survival are not associated with macrophage infiltration in nephrectomy specimens. Figure S3. Overall survival of stage I RCC patients by IRF8 expression in nephrectomy specimens. Figure S4. CD68+ TAM infiltration is unchanged in metastasis expressing high and low levels of IRF8. Figure S5. CD3 T cell infiltration in metastatic RCC tumors. (PDF 563 kb) [file 40425_2019_630_MOESM1_ESM.pdf]

Muhitch et al.
Supplementary Fig. 1

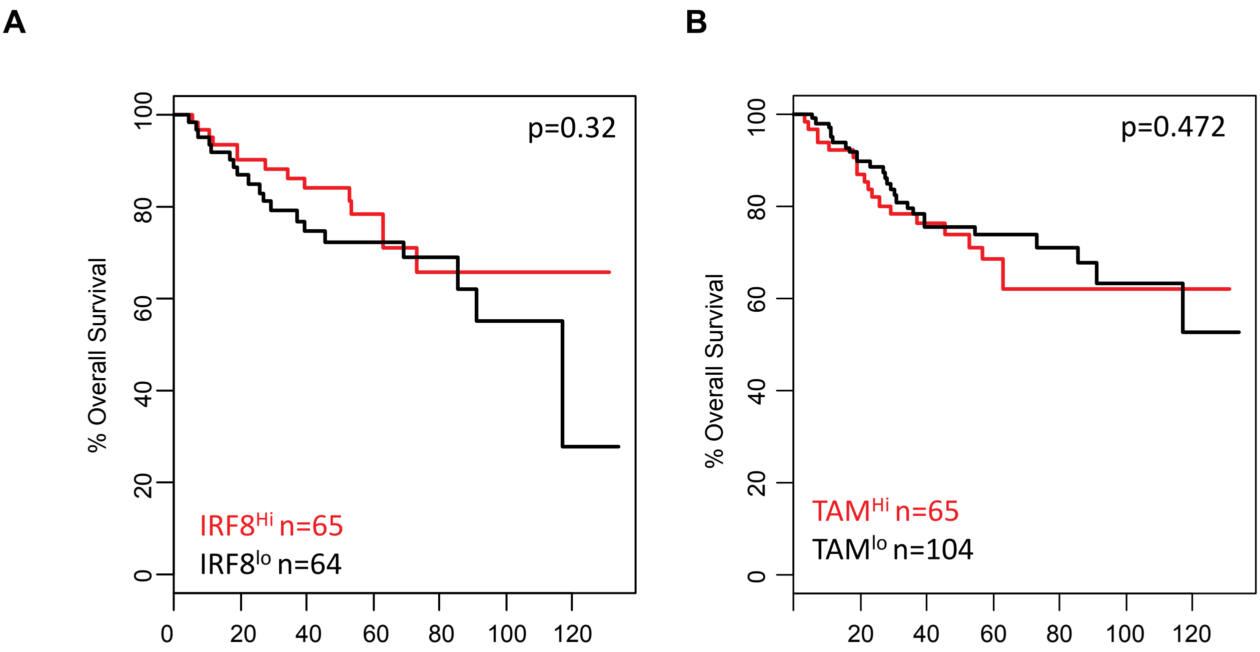

Supplementary Figure S1. Survival of RCC patients by IRF8 and TAM infiltration. Data for overall survival are shown according to stratification based on (A) IRF8 transcript levels or (B) TAM infiltration within primary tumors of RCC patients (panels A and B, respectively). Comparisons performed by log-rank test.

Muhitch et al.
Supplementary Fig. 2

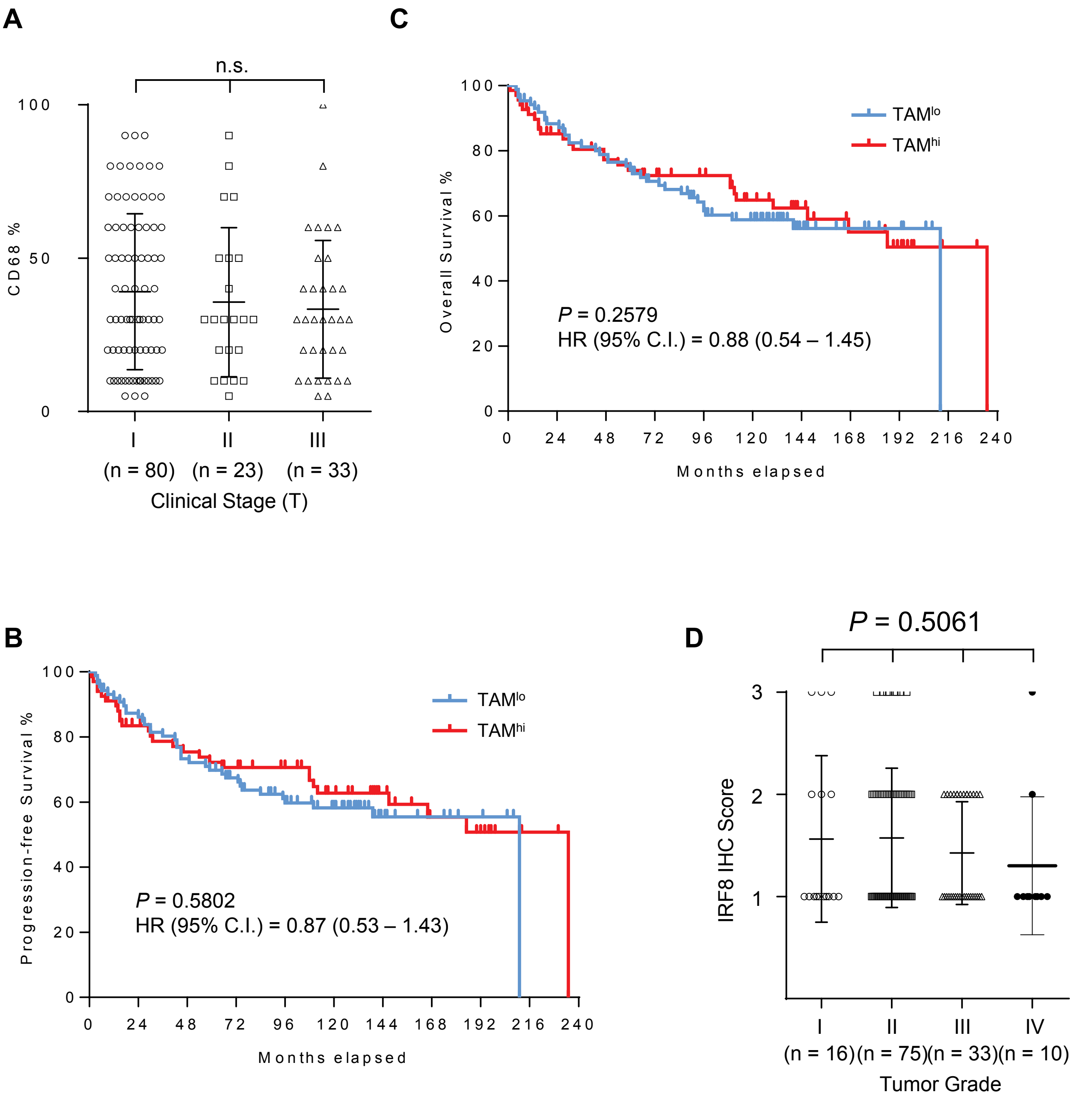

Supplementary Figure S2. Stage, progression-free and overall survival are not associated with macrophage infiltration in nephrectomy specimens. (A) Percentage of $CD68^{+}$ TAM per histological section plotted by clinical stage (T). Comparison performed by Kruskal-Wallis test. Data for progression-free survival and overall survival are shown according to stratification based on CD68 infiltration within primary tumors of patients (panels B and C, respectively). No significant differences in survival were observed for patients with high levels of intratumoral macrophages (> 30% TAM) compared to RCC patients with low levels of TAM (≤ 30%) based on log-rank test. (D) IRF8 intensity (IHC score) plotted by tumor grade, comparison performed by Kruskal-Wallis test. .

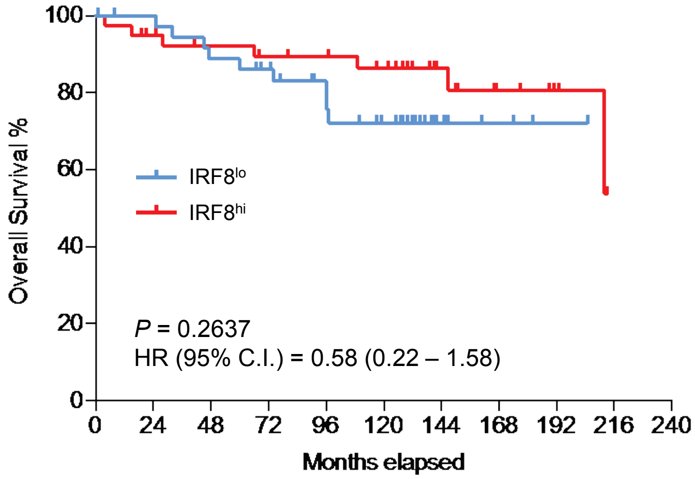

Supplementary Figure S3. Overall survival of stage I RCC patients by IRF8 expression in nephrectomy specimens. Data for overall survival are shown according to stratification based on IRF8 status within primary tumors of stage I RCC patients.

Muhitch et al.
Supplementary Fig. 4

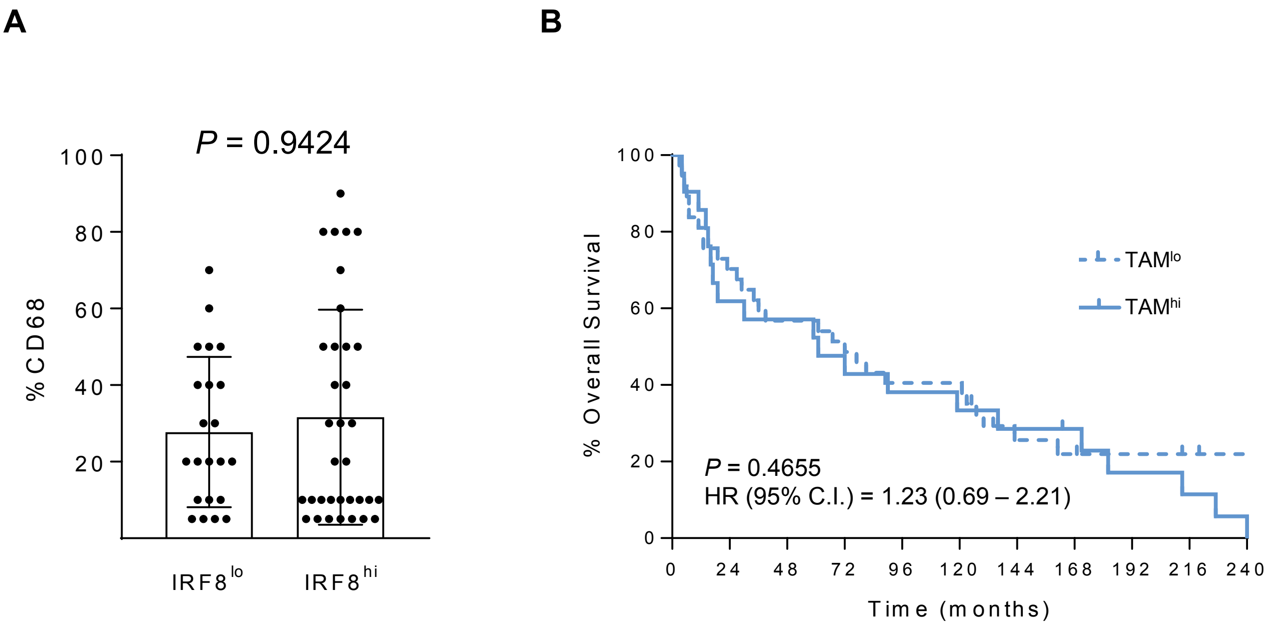

Supplementary Figure S4. CD68+ TAM infiltration is unchanged in metastasis expressing high and low levels of IRF8. (A) Percentage of CD68+ TAM per histological section are plotted by IRF8 low and IRF8 high status (< 2 and ≥ 2 respectively). Comparison performed by unpaired Mann-Whitney test. (B) Data for overall survival are shown according to stratification based on CD68 infiltration within metastatic tumors of patients. No significant differences in survival were observed for patients with high levels of intratumoral macrophages (> 30% TAM) compared to RCC patients with low levels of TAM (≤ 30%) based on log-rank test.

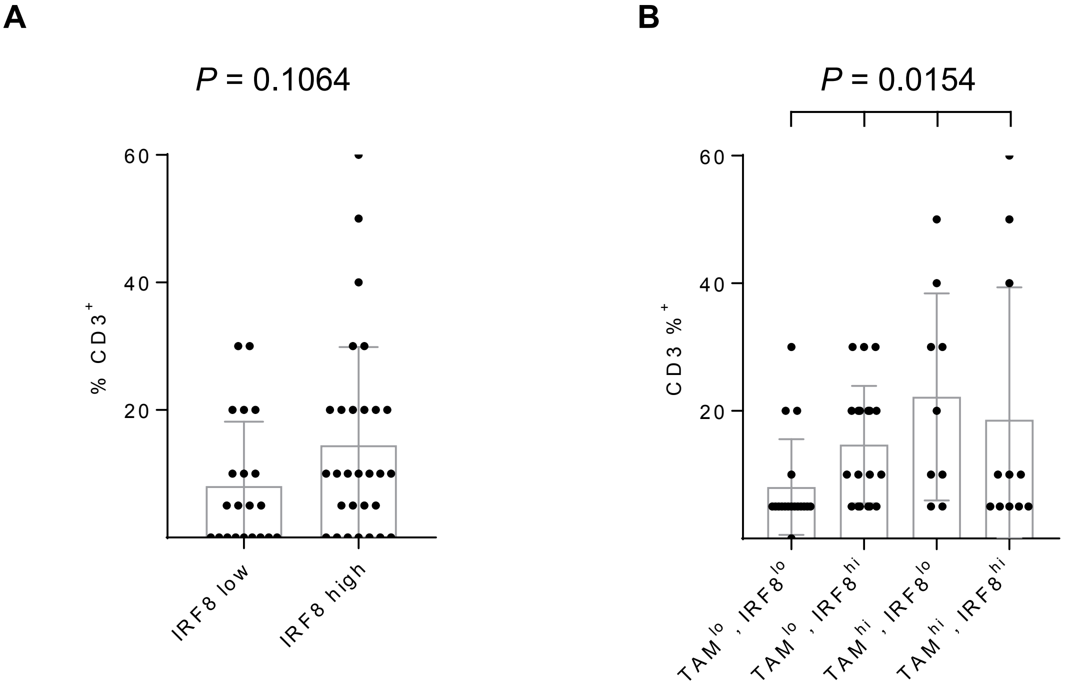

Supplementary Figure 5. CD3 T cell infiltration in metastatic RCC tumors. (A) CD3 T cell infiltration is unchanged in metastasis expressing high and low levels of IRF8. Comparison performed by unpaired Mann-Whitney test. (B) Percentage of $CD3^+$ T cells per histological section are plotted by $CD68^+$ TAM and IRF8 status (< 2 and ≥ 2 respectively). Comparisons performed by Kruskal-Wallis test.
